# The small RNA family gets bigger: AGO1-bound noncanonical sRNAs are upregulated under nutrient deprivation in *Chlamydomonas*

**DOI:** 10.1093/plcell/koad092

**Published:** 2023-03-27

**Authors:** Johan Zicola

**Affiliations:** Assistant Features Editor, The Plant Cell, American Society of Plant Biologists, USA; Department of Crop Sciences and Center for Integrated Breeding Research, The University of Göttingen, Göttingen, Low Saxony 37075, Germany

Small RNAs (sRNAs) are non-coding RNAs of less than 200 nucleotides (nt) in length. They can silence gene expression posttranscriptionally via RNA interference or transcriptionally via DNA methylation. In plants, most sRNAs are derived from dsRNA cleaved by DICER LIKE (DCL) proteins and are 20 to 24 nt in length. ARGONAUTE (AGO) proteins load sRNA duplexes with an affinity depending on the duplex structure, length, and 5′ nucleotide composition. AGOs and sRNAs form the RNA-induced silencing (RISC) complex essential to sRNA regulatory functions. *Chlamydomonas reinhardtii*, a unicellular alga, possesses 3 DCLs (DCL1/2/3) and 3 AGOs (AGO1/2/3), with a known function only assigned to AGO3 and DCL3 so far (Voshall et al. 2015; Valli et al. 2016).

In new work, Li et al. (2023) show that AGO1 in *C. reinhardtii* preferentially binds sRNAs longer than >26 nt and that the expression of these sRNAs is induced under sulfur and nitrogen deprivation. The upregulation of these sRNAs coincides with the downregulation of expression of genes sharing sequence similarity, indicating a potential role of these sRNAs in nutrient stress response.

The authors started their investigation by comparing published sRNA sequencing data from different species of algae and plants. They found a clear enrichment of sRNAs longer than 26 nt in *C. reinhardtii*. In contrast to *C. reinhardtii*, the alga *Volvox carteri* does not show this enrichment and lost its AGO1 ortholog. To assess whether the >26 nt sRNAs are associated with AGO1 in *C. reinhardtii*, the authors generated transgenic algae producing FLAG-tagged AGO1 proteins that can be purified to extract the sRNAs they contain and also used a transgenic line producing FLAG-tagged AGO3 protein. They show that AGO3 and AGO1 bind preferentially 20 to 22 nt and >26 nt sRNAs, respectively (Figure 1).

**Figure 1.**
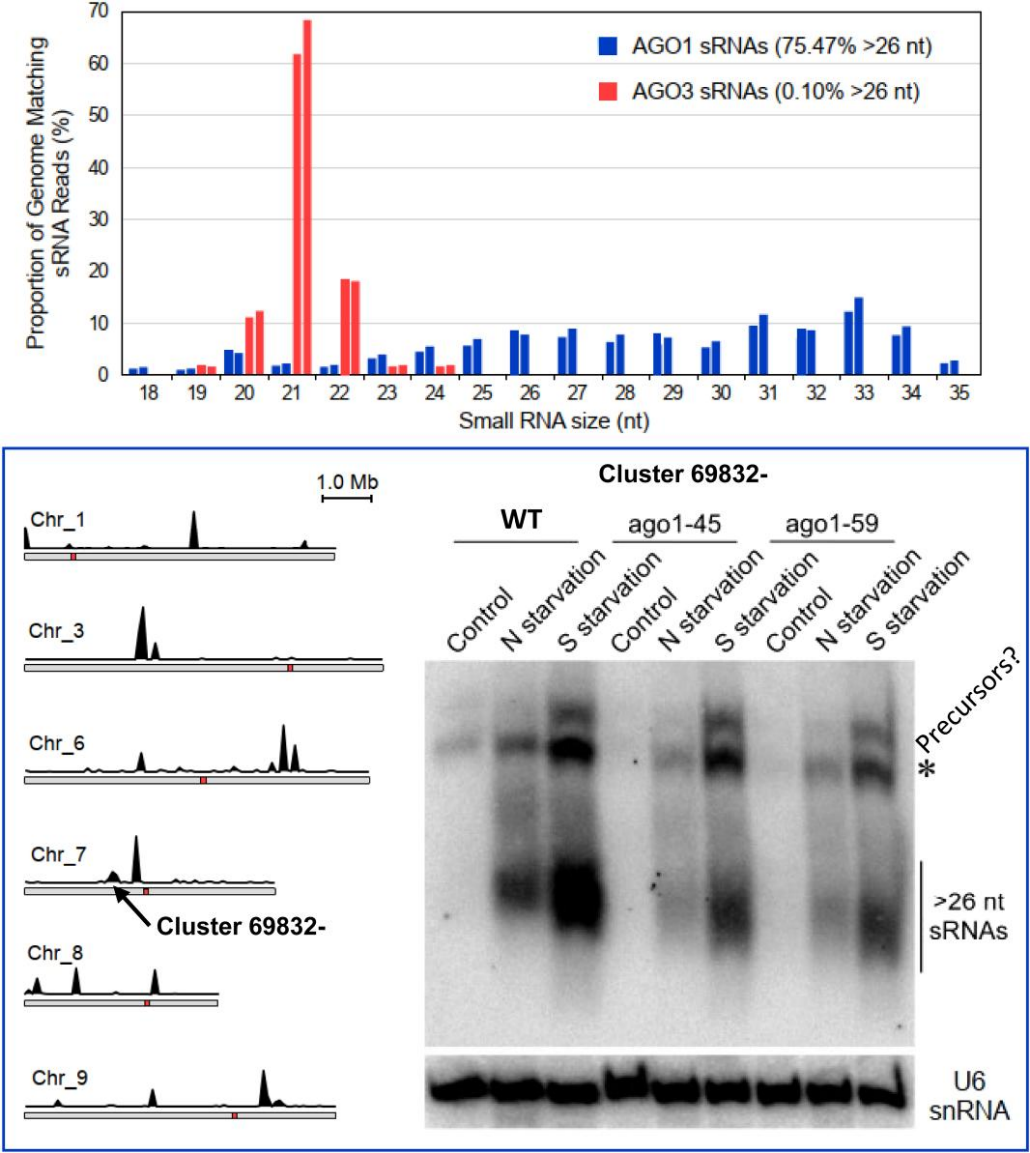
Small RNAs longer than 26 nt are bound by AGO1, map to distinct clusters in the genome, and are upregulated under nitrogen (N) and sulfur (S) deprivation. Their level is reduced in *ago1* knockout mutants. Size distribution of genome mapped AGO1-associated sRNAs and AGO3-associated Maa7-IR44 CC-124 Maa7-IR44sRNAs. Replicate sRNA libraries are shown with bars of the same color (top). Location of genomic loci encoding AGO1-associated >26 nt sRNAs on *Chlamydomonas* chromosomes (bottom left) and immunoblot analyses of small RNAs isolated from the indicated strains and detected with probes specific for the most abundant >26 nt sRNAs (bottom right). Adapted from Li et al. (2023, Figures 2, 3, and 4).

The team found that AGO1-bound sRNAs are derived from dozens of moderately repetitive clusters across the genome (Figure 1). The authors predicted hairpin RNA precursors from the sRNA clusters with structures reminiscent of primary miRNAs. Using northern blot probes for 3 of these clusters, they found that the >26 nt sRNAs are strongly upregulated when the alga is grown under nitrogen or sulfur deprivation (Figure 1). They could also observe a comparable nutrient-mediated upregulation of >26 nt sRNAs in another *Chlamydomonas* species for 3 clusters, indicating a conserved mechanism across related species.

The authors next examined the level of >26 sRNAs in *dcl1/2/3* mutants. They found that the production of >26 nt sRNAs was similar or increasing in the 3 mutants compared with wild type, suggesting that their biogenesis is independent from DCL1/2/3 activity. They then focused on 1 >26 nt sRNA cluster that falls within the intron of a gene possibly involved in iron–sulfur metabolism and found that upon nutrient deprivation, the gene is downregulated. In the *ago1* Cas9 mutants grown under nutrient deprivation, the level of >26 nt sRNAs of this cluster was reduced, and the expression of the gene was slightly higher than in the wild type, supporting a regulatory function of AGO1-bound >26-nt sRNAs at this locus. The >26-nt sRNAs of the locus were also predicted to target 32 other genes based on sequence similarity. These sRNAs may therefore regulate a total of 33 genes.

Based on published transcriptomic data, half of the 33 genes are downregulated under nutrient deprivation. Interestingly, the authors could not detect any cleavage of the predicted target mRNAs within the >26 nt sRNA binding sequence, indicating that further work is needed to investigate their potential mode of action. This study highlights a new regulatory class of sRNAs that may also be present in plants, although at a much lower level than in *C. reinhardtii*. Their exact action mechanism and potential regulatory functions in other species will hopefully foster exciting discoveries.

## References

[koad092-B1] Li Y , Kim E-J, Voshall A, Moriyama EN, Cerutti H. Small RNAs >26 nt in length associate with AGO1 and are upregulated by nutrient deprivation in the alga Chlamydomonas. Plant Cell. 2023:35(6):1868–1887. 10.1093/plcell/koad093 36945744 PMC10226591

[koad092-B2] Valli AA , Santos BACM, Hnatova S, Bassett AR, Molnar A, Chung BY, Baulcombe DC. Most microRNAs in the single-cell alga *Chlamydomonas reinhardtii* are produced by Dicer-like 3-mediated cleavage of introns and untranslated regions of coding RNAs. Genome Res. 2016:26(4):519–529. 10.1101/gr.199703.115 26968199 PMC4817775

[koad092-B3] Voshall A , Kim E-J, Ma X, Moriyama EN, Cerutti H. Identification of AGO3-associated miRNAs and computational prediction of their targets in the green alga *Chlamydomonas reinhardtii*. Genetics. 2015:200(1):105–121. 10.1534/genetics.115.174797 25769981 PMC4423357

